# Presynaptic Autophagy and the Connection With Neurotransmission

**DOI:** 10.3389/fcell.2021.790721

**Published:** 2021-12-17

**Authors:** Marianna Decet, Patrik Verstreken

**Affiliations:** ^1^ VIB-KU Leuven Center for Brain & Disease Research, Leuven, Belgium; ^2^ KU Leuven, Department of Neurosciences, Leuven Brain Institute, Mission Lucidity, Leuven, Belgium

**Keywords:** macroautophagy, synapse, synaptic autophagy, neurotransmission, vesicle cycling

## Abstract

Autophagy is an evolutionary conserved catabolic pathway essential for the maintenance of cellular homeostasis. Defective proteins and organelles are engulfed by autophagosomal membranes which fuse with lysosomes for cargo degradation. In neurons, the orchestrated progression of autophagosome formation and maturation occurs in distinct subcellular compartments. For synapses, the distance from the soma and the oxidative stress generated during intense neuronal activity pose a challenge to maintain protein homeostasis. Autophagy constitutes a crucial mechanism for proper functioning of this unique and vulnerable cellular compartment. We are now beginning to understand how autophagy is regulated at pre-synaptic terminals and how this pathway, when imbalanced, impacts on synaptic function and -ultimately- neuronal survival. We review here the current state of the art of “synaptic autophagy”, with an emphasis on the biogenesis of autophagosomes at the pre-synaptic compartment. We provide an overview of the existing knowledge on the signals inducing autophagy at synapses, highlight the interplay between autophagy and neurotransmission, and provide perspectives for future research.

## Introduction

Macroautophagy, hereafter ‘autophagy’, is a conserved, regulated pathway that cells use to turn-over organelles and proteins, and create new bio-molecules. Damaged material is engulfed by a double-membrane and directed to lysosomes for hydrolytic degradation.

In the early 1990’s, Yoshinori Ohsumi was the first to characterize the genes essential for autophagy (Tsukada and Ohsumi, 1993). By now, more than 30 autophagy (Atg) related genes, instructing every step of autophagosome biogenesis, have been characterized, mostly in studies performed in yeast (reviewed in (Nakatogawa, 2020)).

Like other cell types, neurons employ autophagy to maintain homeostasis and ensure survival. This is particularly important because most neurons are not regenerated during adult life and they need to maintain functional over many decades. Furthermore, autophagy also serves a more direct role in neuronal physiology, like neurotransmission (Kuijpers et al., 2021), synaptic plasticity (Compans et al., 2021; Pan et al., 2021), circuit development (Tang et al., 2014; Linda et al., 2021; Zapata-Muñoz et al., 2021) and survival (Hara et al., 2006; Komatsu et al., 2006; Komatsu et al., 2007). Moreover, the spatial organization of autophagy within neurons is unique. A growing body of literature, including work from our lab, shows that neuronal autophagy is highly compartmentalized (Maday and Holzbaur, 2016; Soukup et al., 2016; Okerlund et al., 2017; Vanhauwaert et al., 2017; Kulkarni et al., 2021), reflecting the peculiar morphology of neurons. In this review, we focus on the biogenesis of autophagosomes at the pre-synaptic compartment and on the inter-connection between autophagy and synaptic transmission.

## Synaptic Autophagy

The unique physiology of synapses makes them susceptible to protein and organelle damage. Synaptic transmission is an energy demanding process. The production of energy by mitochondria results in the accumulation of reactive oxygen species (ROS), which might affect protein functionality. Additionally, synapses are often located far from the soma. Although local translation in distal axons of mature neurons has been reported (Hafner et al., 2019), other synaptic components are synthetized in the cell body and transported along the axon (Goldstein et al., 2008). The combination of these factors suggests the need for synapses to locally regulate the turnover of their proteome and organelles as a way to maintain homeostasis.

Consistent with this hypothesis, several studies performed in cultured neurons showed the presence of ATG8/LC3 positive structures at distal axons, a commonly used marker for autophagosomes, indicating that autophagosome formation occurs at this location (Maday et al., 2012; Wang et al., 2015; Okerlund et al., 2017). Parallel work in *Drosophila* neuromuscular junction (NMJ) synapses showed with higher spatial resolution that autophagosome biogenesis takes place at the pre-synaptic compartment (Soukup et al., 2016; Vanhauwaert et al., 2017).

Autophagosome formation in neurons proceeds in a step-wise manner (Maday and Holzbaur, 2016). Interestingly, in addition to the classical ATG proteins, some of the proteins constituting the synaptic machinery and driving synaptic vesicle (SV) cycling have a function in autophagy. The scaffold protein Bassoon (tom Dieck et al., 1998) has been shown to inhibit autophagosome formation through ATG5 (presented in detail below (Okerlund et al., 2017)), while the endocytic proteins Endophilin-A (EndoA) and Synaptojanin1 (Synj1) (Verstreken et al., 2002; .Verstreken et al., 2003) have the opposite function. By acting directly on autophagosomal membranes, EndoA and Synj1 have a positive effect on synaptic autophagosome formation (Soukup et al., 2016; Vanhauwaert et al., 2017). Interestingly, these proteins are strongly enriched at synapses and autophagy in neuronal cell bodies is not affected, indicating synaptic autophagosome formation is regulated in a compartment-specific manner.

Following their biogenesis, autophagosomes are retrogradely transported along the axon by dynein motor proteins (Ravikumar et al., 2005). During transport, autophagosomes become increasingly acidic by fusing with lysosomes (Maday et al., 2012), as detected by imaging of the tandem reporter GFP-mCherry-ATG8/LC3 (Kimura et al., 2007). However, acidification of autophagosomes prior to transport has been observed at *Drosophila* NMJs (Vanhauwaert et al., 2017), suggesting that the fusion with lysosomes is not strictly linked to transport. Defects in the fusion and transport of autolysosomes, therefore in the cargo clearance, impact neuronal physiology and can result in neurodevelopmental or neurodegenerative disorders (Lee et al., 2011; Hori et al., 2017; Tammineni and Cai, 2017; Wang et al., 2018). For more details on autophagosomes transport and maturation we refer to a thorough review by Hill and Colón-Ramos (Hill and Colón-Ramos, 2020).

**FIGURE 1:**
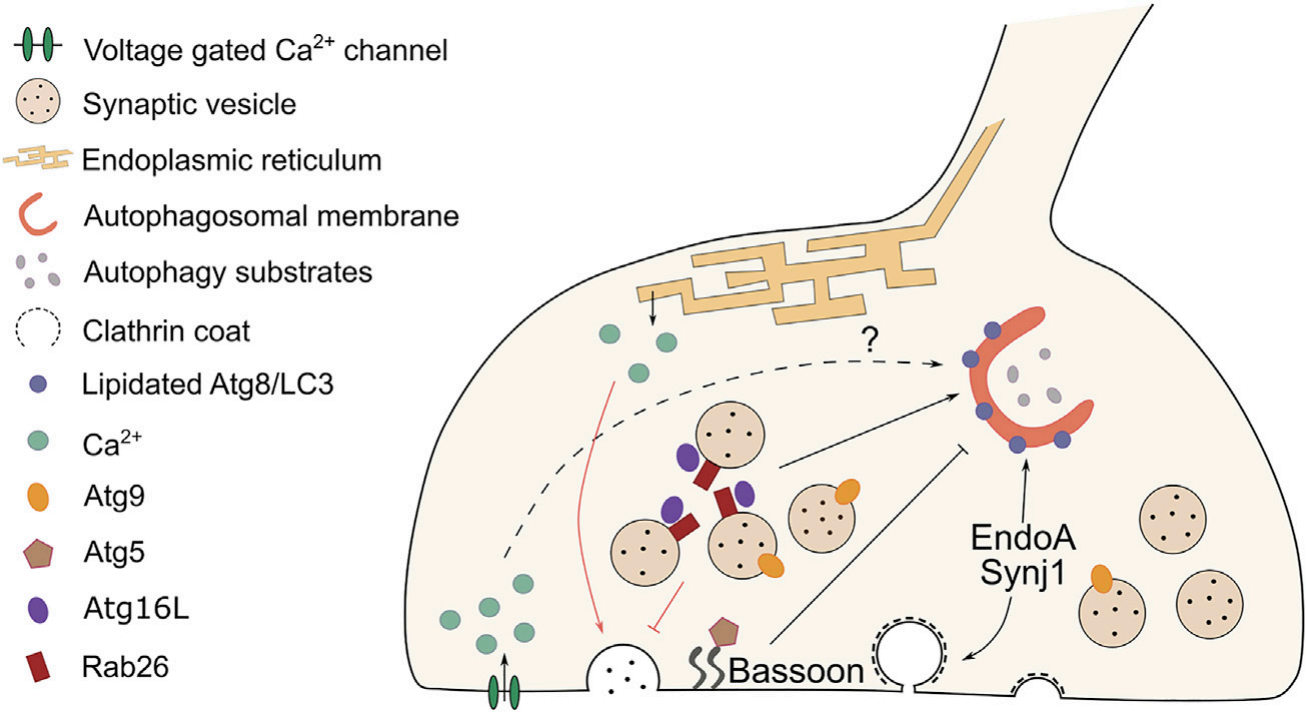
Cross-regulation between autophagy and synaptic vesicle cycling at the pre-synaptic terminal. At synapses, synaptic vesicle (SV) cycling and autophagy are interconnected and reciprocally regulated. Black lines indicate the effect on autophagosome formation elicited by components of the SV machinery, while red lines highlight the influence on neurotransmission evoked by changes in autophagy levels. The endocytic proteins EndoA and Synj1 participate in autophagosome formation respectively by recruiting ATG3 and by allowing ATG18 to cycle off the autophagosomal membranes. Both actions result in the lipidation of ATG8/LC3. The scaffold protein Bassoon has the opposite effect. It sequesters ATG5 and prevents autophagosome formation. Pre-synaptic calcium waves are associated with autophagy induction, but a direct mechanistic link between the two is missing. Cycling of ATG9-positive vesicles facilitate autophagosome formation at pre-synaptic sites. The GTPase Rab26 clusters on SVs and by interacting with ATG16L it recruits the autophagy machinery that engulfs and degrades SVs. SV turnover modulates the strength of neurotransmission. Reduced numbers of SVs, due to autophagy-mediated clearance, results in decreased neurotransmitter release. An opposite effect is observed when autophagy is abolished and tubular ER accumulates at synapses. As a consequence, calcium buffering is altered and neurotransmission is enhanced. The crosstalk between synaptic autophagy and neurotransmission might represent a feedback mechanism that synapses employ to regulate both pathways in a flexible way.

Growing evidence shows that synaptic autophagy and neurotransmission are reciprocally regulated, but the mode and the functional implications for this crosstalk are not fully understood. Here, we provide an overview of the current knowledge on the interconnection between SV cycling and autophagy at synapses (Figure 1).

## Neuron-specific Signals for Autophagy Induction: How Neurotransmission Regulates Synaptic Autophagy

Cultured neurons display basal autophagy in distal axons (Maday et al., 2012; Maday and Holzbaur, 2016), suggesting that autophagosomes are constitutively formed at this location. Neuronal autophagy can be further triggered by a plethora of signals. Nutrient deprivation is a classical way of inducing non-selective autophagy, as it has been shown to increase the level of autophagosomal markers in multiple tissues *in vivo* (Mizushima et al., 2004). Starvation proved to be an effective, but not neuron-specific, way to induce autophagy in *Drosophila* NMJs (Soukup et al., 2016; Vanhauwaert et al., 2017), in distal axons of primary neuronal culture (Young et al., 2009; Murdoch et al., 2016) and in neurites of iPSCs derived neurons (Vanhauwaert et al., 2017).

Local autophagy levels in neurons are modulated by synaptic activity. Increased firing rate of AIY interneurons in *C. elegans* stimulate autophagosome formation near synapses, an effect abolished by genetic inhibition of neurotransmission (Hill et al., 2019). Similarly, electrical stimulation of motor neurons and activation of the temperature-sensitive TrpA1 channel induce autophagosome biogenesis at pre-synaptic terminals of *Drosophila* NMJs (Soukup et al., 2016). Moreover, prolonged KCl-mediated depolarization of cultured hippocampal neurons increases the number of ATG8/LC3 positive autophagosomes in axon terminals (Wang et al., 2015), but also in cell soma and dendritic spines (Shehata et al., 2012). However, the molecular mechanisms that connect neuronal stimulation to the induction of autophagy at synapses is elusive.

KCl stimulation does not only induce autophagosome biogenesis at pre- and post-synaptic sites, but also impacts the retrograde trafficking of autophagic vacuoles along axons of cultured neurons (Wang et al., 2015). In contrast, treatment with KCl and K^+^ channel agonists results in decreased mobility of LC3 positive vacuoles in dendrites (Kulkarni et al., 2021). Furthermore, application on cultured neurons of the glutamate analog NMDA increases the number of axonal autophagosomes and this also stimulates the movement of a portion of these autophagosomes (Katsumata et al., 2010). NMDA treatment is also associated with long term depression (LTD), a form of synaptic plasticity in which dendritic spines are pruned, in this case by increased autophagy (Shehata et al., 2012; Compans et al., 2021). These observations suggest that while both the pre- and post-synapse respond to neuronal activity by promoting autophagosome formation, the dynamics of newly formed autophagosomes might be differentially regulated among synaptic compartments.

Interestingly, not single action potentials but rather prolonged neuronal stimulation has been shown to increase the number of autophagosomes at synapses (Soukup et al., 2016; Vanhauwaert et al., 2017). Synaptic protein homeostasis is challenged during high firing rates, which could explain why autophagy is only upregulated during intense neuronal activity. We speculate that neuronal activity-induced autophagy is a form of selective autophagy aimed at recycling (damaged) synaptic material. In line with this, a study in *C. elegans* showed that autophagic vacuoles that formed upon activity-dependent autophagy contain synaptic proteins (Hill et al., 2019). This also raises the question of whether basal autophagy levels are different among neuron subtypes that physiologically show distinct activity patterns. If so, this might contribute to the selective neuronal vulnerability to degeneration.

The modulation of autophagy by prolonged synaptic activity is mostly unexplored. While the effect of intracellular calcium on autophagosome formation is still under debate (Bootman et al., 2018), it would be interesting to evaluate the role of activity-induced calcium influx at the synapse, which could potentially function as a messenger for autophagy induction during neuronal stimulation. Activity-dependent calcium waves have been associated to autophagosome formation at the AIY interneurons in *C. elegans* (Hill et al., 2019). However, whether calcium itself, or other synaptic signals, trigger autophagy has not been investigated.

## How Synaptic Autophagy Modulates Neurotransmission

Modulation of neurotransmission by synaptic autophagy was recently shown to be dependent on calcium release from endoplasmic reticulum (ER) stores (Kuijpers et al., 2021). Specifically, knock out of the core autophagy protein ATG5 results in increased excitatory neurotransmission in acute hippocampal slices and primary culture, an effect independent from synaptic density or SVs number. Neurotransmission is facilitated by cytoplasmic buildup of calcium released from tubular ER, which significantly accumulates at pre-synaptic sites as a consequence of abolished autophagy. In fact, in this study the authors found that tubular ER is a major substrate of synaptic autophagy further highlighting the crosstalk between these two processes (Kuijpers et al., 2021).

While impaired ATG5-dependent autophagy leads to facilitated excitatory neurotransmission *via* altered ER calcium buffering, several studies have reported the opposite effect upon autophagy induction (Hernandez et al., 2012; Binotti et al., 2015; Okerlund et al., 2017). In murine cortico-striatal slices, induction of autophagy by treatment with the mTOR inhibitor rapamycin leads to a drop in the number of SVs and a consecutive decrease in evoked neurotransmitter release, an effect dependent on ATG7 expression and independent from signals from the soma (Hernandez et al., 2012). Loss of SVs has also been observed in cultured neurons in which autophagy was upregulated by double knock down of Bassoon and Piccolo (Okerlund et al., 2017). A potential connecting element between SV turnover and autophagy could be the GTPase Rab26 which has been shown to cluster SVs at *Drosophila* NMJs, and in neurites and soma of cultured hippocampal neurons (Binotti et al., 2015). Interestingly, GST-bound Rab26 interacts with the autophagy protein ATG16L that ultimately recruits ATG8/LC3 to the clustered vesicles, suggesting a role for Rab26 as a receptor for selective autophagy of SVs. This function was shown to be dependent on the activation of Rab26 by the guanine exchange factor Plekhg5 (Lüningschrör et al., 2017). These studies suggest that autophagy modulates neurotransmission by reducing the number of SVs available for release. This effect may be a combination of two aspects: 1) the regulation by autophagy of SVs and SV-associated protein turnover, which are recycled after several rounds of exo-endocytosis (Binotti et al., 2015; Hill et al., 2019), and 2) the use of SVs as membrane source for nascent autophagosomes. In accordance with the latter, electron microscopy analysis of DAB-labeled Bassoon knock-down neurons expressing VAMP2-HRP shows the presence of electron dense deposits of VAMP2-HRP on structures resembling autophagosomes (Okerlund et al., 2017).

An additional putative link between SVs cycling and autophagy is the transmembrane protein ATG9. ATG9, which is present in a subset of SVs (Taoufiq et al., 2020), is a lipid scramblase that mediates the expansion of pre-autophagosomal vesicles by aiding ATG2 in the transfer of lipids from other membrane sources (Matoba et al., 2020; Sawa-Makarska et al., 2020). Recent work in *C. elegans* shows that ATG9 undergoes exo-endocytosis cycles and that disruption of ATG9 trafficking impairs activity-dependent synaptic autophagy (Yang et al., 2020; Xuan et al., 2021). Whether ATG9-containing SVs serve solely as membrane source for autophagosome formation or if ATG9 cycling itself is a signal for autophagy induction remains unknown.

Overall, these studies indicate that pre-synaptic autophagy modulates the strength of neurotransmission by regulating SV turnover (Hernandez et al., 2012; Binotti et al., 2015; Okerlund et al., 2017) and intracellular calcium buffering (Kuijpers et al., 2021). On the other hand, SV cycling can also regulate activity-dependent synaptic autophagy (Yang et al., 2020; Xuan et al., 2021).

It is interesting to note that impaired autophagy does not always lead to alteration in exo-endocytosis cycles. For example, mutant EndoA and Synj1 impact autophagosome formation at glutamatergic *Drosophila* NMJs without obviously affecting basic neurotransmission measurements (Soukup et al., 2016; Vanhauwaert et al., 2017). The reciprocal regulation of autophagy and SV cycling might be different in different neuronal subtypes, or defects in autophagy only manifest under specific conditions (eg stress, remodeling, growth etc). Further studies are required to clarify this.

## Sharing Drivers: Autophagosome Biogenesis at the Pre-synaptic Compartment

The involvement of synapse-resident proteins in both SV cycling and in autophagy is what distinguishes autophagosome formation at pre-synaptic sites from that occurring in other neuronal compartments. Several studies in recent years have shed light onto the molecular mechanism regulating this pathway at synapses, but many questions remain to be addressed in future work. In this section we present these dual functions of Bassoon, EndoA1 and Synj1 in neurotransmission and autophagosome formation.

### Bassoon

Okerlund and colleagues have shown that double knock down of the active zone scaffold proteins Bassoon (tom Dieck et al., 1998) and Piccolo (Cases-Langhoff et al., 1996) results in an increased number of LC3 positive autophagosomes in primary hippocampal cultures (Okerlund et al., 2017). The co-localization between LC3 and VGlut1 or Synaptophysin confirms that the observed autophagosomes are at pre-synaptic sites. This effect is mediated by the interaction between the coiled-coil 2 (CC2) domain of Bassoon with the E3 ubiquitin ligase ATG5, in a region essential for the binding of ATG5 with ATG16L (Zhao et al., 2013). The function of ATG5 is crucial for the lipidation of ATG8/LC3 and its recruitment to autophagosomal membranes (Hara et al., 2006). The results of this study suggest that Bassoon sequesters ATG5, by directly binding to it, thus depleting the protein at synapses and resulting in the inhibition of synaptic autophagy. However, there are ways to induce autophagy in the presence of Bassoon, indicating that signals triggering this pathway overcome the negative effect of Bassoon on autophagy induction. This observation raises several questions: is the function of Bassoon to maintain a low basal autophagy level at synapses? Do other synapse-enriched proteins contribute to this function?

### Endophilin-A1

EndoA1 is well-characterized for its role in SV endocytosis (Ringstad et al., 1997; Verstreken et al., 2002; Milosevic et al., 2011), but more recently an additional function for this protein in autophagy has been found (Murdoch et al., 2016; Soukup et al., 2016).

Work from our group demonstrated, by combining imaging of *Drosophila* NMJs and biochemical assays, that EndoA participates in synaptic autophagy by recruiting ATG3 onto nascent autophagosomes. This effect is mediated by the kinase LRRK2, which phosphorylates EndoA at Serine75 (Matta et al., 2012; Ambroso et al., 2014) in response to starvation. S75 resides on the amphiphilic helix H1 of EndoA BAR domain, which mediates the interaction of EndoA with autophagosomal membranes. Upon phosphorylation, helix H1 inserts less deeply into the lipid bilayer forming highly curved membranes and allowing the recruitment of the curvature-sensing protein ATG3. Ultimately, ATG3 participates in the lipidation of ATG8/LC3 (Nath et al., 2014).

The work of Murdoch et al. has shown that the role of EndoA in synaptic autophagy is conserved in mice (Murdoch et al., 2016). Triple knock outs for EndoA1, two and three show significantly decreased ATG8/LC3 lipidation and increased level of ubiquitinated proteins. The authors propose that the function of EndoA on autophagy is linked to FBXO32 levels, which is also associated with autophagy induction (Murdoch et al., 2016). Further work on how EndoA and FBXO32 cooperate in this process is however still missing. Furthermore, what is also still poorly understood is the relationship between endocytosis and autophagy at synapses. As EndoA phospho-mutants do not affect SVs cycling (Soukup et al., 2016), is the function of EndoA in autophagy fully independent from endocytosis? The modalities by which EndoA switches from one function to the other or couples these two synaptic pathways also require further investigation.

### Synaptojanin-1

Synj1 contains two lipid phosphatase domains: a 5’ phosphatase and a SAC1 domains. Both have been implicated in autophagosome formation (George et al., 2016; Vanhauwaert et al., 2017).

Synj1 deficient zebrafish has been shown to accumulate late endosomes and immature autophagosomes in cone photoreceptors, suggesting a role for Synj1 in autophagosome maturation (George et al., 2016). This effect is mediated by the activity of the 5’ phosphatase domain, but not of the SAC1 domain. On the other hand, Vanhauwaert and colleagues show a function for the latter domain on early phases of autophagosome formation. Specifically, a mutation in the SAC1 domain also found in Parkinson patients, inhibits the dephosphorylation of PI(3)P/PI(3,5)P_2_ on autophagosomal membranes, resulting in the accumulation of the PI(3)P-binding protein ATG18a/WIPI2 on the membrane of nascent autophagosomes, and the consecutive failure in ATG8/LC3 recruitment (Vanhauwaert et al., 2017). Complementary work in primary hippocampal culture and *C. elegans* has shown that the Parkinson-associated mutation in Synj1 SAC1 domain affects the localization of ATG9, which abnormally accumulates in subsynaptic foci (Yang et al., 2020). In line with these studies, a recent work on a *Synj1* haploinsufficiency mouse model reports the accumulation of insoluble p62, an autophagy substrate, in brain of aged mice, suggesting that autophagy is impaired (Pan et al., 2020).

Taken together, these studies provide evidence for a role of Synj1 in promoting synaptic autophagy, with each lipid phosphatase domain contributing to a different step of the pathway, although the latter aspect needs to be further explored.

It is worth noting that EndoA and Synj1 mutants that inhibit autophagy induction at synapses show a certain level of residual autophagy, as a number of ATG8/LC3 puncta is still detected (Soukup et al., 2016; Vanhauwaert et al., 2017). This observation could be explained by the existence of two independent pathways controlling autophagy at synapses, where one EndoA/Synj1-independent pathway maintains basal autophagy, and one synapse-specific cascade responds to “synaptic triggers” and “insults”. The coexistence of those pathways could mediate a fine regulation of autophagy that synapses likely need in comparison to other neuronal compartments or cell types. In fact, the anatomical and physiological features of synapses challenge them in the maintenance of a healthy proteome throughout the entire life of an organism.

## Conclusion and Open Questions

Converging lines of research have shed light onto the mechanism of autophagosome biogenesis at distal axons and synapses and the interactions with neurotransmission. The synapse-enriched proteins Bassoon, EndoA1 and Synj1 actively modulate autophagosome formation at those locations (George et al., 2016; Murdoch et al., 2016; Soukup et al., 2016; Okerlund et al., 2017; Vanhauwaert et al., 2017). Their role in SV cycling place them in an ideal position to cross-regulate those two pathways. Although there is no direct evidence yet on how those proteins achieve this dual function, it is tempting to speculate that they might respond to a unique signal triggering both pathways, eg calcium, voltage changes, cytoskeletal elements or others, but a direct mechanistic link is still missing (Hill et al., 2019). Investigating the substrates of autophagy depending on the initial trigger could provide a deeper understanding of how this pathway is regulated at synapses. It would be interesting to verify the existence of signal-specific forms of synaptic autophagy that are cargo selective and aimed at maintaining the number and functionality of defined organelles. For example, neuronal activity specifically inducing recycling of SVs, ER stress triggering ER-phagy or localized ROS activating mitophagy. Additionally, the existence of organelle-specific cargo recognition receptors could aid the selectivity of autophagy.

In-depth understanding of the interplay between SV cycling and autophagy, but also the modalities of cargo recognition at synapses, is not only interesting from a pure cell biology perspective, but also crucial from a pathological point of view. A growing body of literature shows that synaptic dysfunction precedes neuronal loss in several neurodegenerative diseases (Delva et al., 2020; Largo-Barrientos et al., 2021; Ravalia et al., 2021). Impaired synaptic function is associated with imbalances in neurotransmission and the accumulation of dysfunctional and aggregated proteins, pointing to defective synaptic homeostasis as underlying mechanism. In support of this hypothesis, numerous risk factors and mutated genes in neurodegenerative diseases encode proteins involved in both endocytic trafficking and autophagy. Examples are PICALM/CALM contributing to Alzheimer’s disease pathology (Moreau et al., 2014), C9ORF72 implicated in ALS and FTD (Farg et al., 2014) and several genes linked to Parkinson’s disease such as LRRK2, VPS35, SNCA, Synj1 and EndoA (Volpicelli-Daley et al., 2014; Zavodszky et al., 2014; Soukup et al., 2016; Vanhauwaert et al., 2017). Altered neurotransmission and autophagy are likely to contribute to the synaptic vulnerability observed in the prodromal phase of neurodegeneration.

The interconnection between autophagy and neurotransmission represents an underexplored research avenue. Promising directions include: 1) to decipher how the cycling of vesicles at synapses signals autophagy induction, 2) to unravel how these two vital pathways are cross-regulated in pathological conditions, 3) to uncover novel synapse-specific modulators of this pathway, 4) to define the cargo, and thus the purpose of synapse-specific induction of autophagy.
